# Supplementary material for: Factors shaping the gender wage gap among college-educated computer science workers
Source: PLoS One. 2023 Oct 30;18(10):e0293300. doi: 10.1371/journal.pone.0293300 (PMC10615266; doi:10.1371/journal.pone.0293300)
Supplement: S1 File — Notes: Data come from the 2009–2019 American Community survey. The sample is restricted to those ages 22 to 60 with a Bachelor’s degree working full time (35+ hours/week) with positive income in a computer science occupation. A computer science occupation is defined by the Census Bureau in Landivar (2013). We exclude those whose youngest child in the household is over 18. Respondents must also have a valid birthyear and received their degree after 1980. Averages are weighted using the ACS person weights. (DOCX) [file pone.0293300.s001.docx]

**Appendix Table A1. Descriptive Statistics by Occupation and Gender**

| **Occupation** | **Female** | **Average Wage** | | | | **Usual Hours** | | | | **Count** | | |
| --- | --- | --- | --- | --- | --- | --- | --- | --- | --- | --- | --- | --- |
|  |  | **All** | **Male** | **Female** | **Gap** | **All** | **Male** | **Female** | **Gap** | **All** | **Male** | **Female** |
| CIS Manager | 0.28 | $44.94 | $47.02 | $39.69 | -$7.33 | 45.34 | 45.69 | 44.43 | -1.26 | 36,868 | 26,240 | 10,628 |
| Computer Scientist | 0.26 | $36.21 | $37.40 | $32.87 | -$4.53 | 43.79 | 44.09 | 42.93 | -1.16 | 4,766 | 3,432 | 1,334 |
| Computer Analyst | 0.36 | $34.69 | $36.75 | $31.03 | -$5.72 | 43.52 | 43.93 | 42.80 | -1.13 | 29,621 | 18,791 | 10,830 |
| Information Analyst | 0.19 | $39.81 | $40.85 | $35.22 | -$5.63 | 43.70 | 43.90 | 42.80 | -1.10 | 4,000 | 3,246 | 754 |
| Programmers | 0.22 | $36.24 | $37.06 | $33.28 | -$3.78 | 42.64 | 42.86 | 41.87 | -0.99 | 25,775 | 20,095 | 5,680 |
| Software Developer | 0.20 | $44.59 | $46.08 | $38.49 | -$7.59 | 42.58 | 42.73 | 41.95 | -0.78 | 29,324 | 23,522 | 5,802 |
| Web Developer | 0.36 | $27.60 | $28.65 | $25.77 | -$2.88 | 42.73 | 43.01 | 42.23 | -0.78 | 8,846 | 5,650 | 3,196 |
| Support Specialist | 0.25 | $28.34 | $29.03 | $26.33 | -$2.70 | 42.45 | 42.56 | 42.12 | -0.44 | 21,778 | 16,132 | 5,646 |
| Database Admin. | 0.32 | $34.88 | $36.86 | $30.74 | -$6.13 | 42.75 | 43.01 | 42.22 | -0.79 | 7,016 | 4,698 | 2,318 |
| Network Admin. | 0.20 | $31.64 | $32.16 | $29.60 | -$2.56 | 43.05 | 43.18 | 42.54 | -0.65 | 11,842 | 9,381 | 2,461 |
| Network Architect | 0.11 | $41.25 | $32.16 | $29.60 | -$2.56 | 44.04 | 44.15 | 43.06 | -1.09 | 4,869 | 4,357 | 512 |
| All Other CS | 0.24 | $33.90 | $34.60 | $31.68 | -$2.93 | 42.86 | 42.98 | 42.48 | -0.51 | 21,941 | 16,592 | 5,349 |
